# Novel reconstruction method using long and narrow gastric tube in laparoscopic proximal gastrectomy for cancer: a retrospective case series study

**DOI:** 10.3389/fsurg.2024.1413939

**Published:** 2024-07-25

**Authors:** Yoshitake Ueda, Takahide Kawasaki, Sanshi Tanabe, Kosuke Suzuki, Shigeo Ninomiya, Tsuyoshi Etoh, Masafumi Inomata, Norio Shiraishi

**Affiliations:** ^1^ Department of Comprehensive Surgery for Community Medicine, Oita University Faculty of Medicine, Oita, Japan; ^2^ Department of Gastroenterological and Pediatric Surgery, Oita University Faculty of Medicine, Oita, Japan

**Keywords:** laparoscopic surgery, proximal gastrectomy, esophagogastrostomy, gastric tube reconstruction, gastric cancer

## Abstract

**Background and objectives:**

To clarify the safety and feasibility of laparoscopic proximal gastrectomy (LPG) with our novel reconstruction methods.

**Methods:**

Novel method is a reconstruction with a long and narrow gastric tube with widening of the proximal side created by linear stapler, and esophagogastrostomy is performed by linear stapler. In conventional method, esophagogastrostomy is performed by a circular stapler. Short- and long-term outcomes of a novel method were compared with those of conventional method.

**Results:**

A total of 44 patients whom LPG was performed were enrolled in this retrospective study. No cases of anastomotic leakage and stenosis were observed in both groups. The cases of postoperative reflux esophagitis (Grade B or higher) at 1 year after operation in the Novel group were less than those in the Conventional group (17% vs. 44%).

**Conclusion:**

LPG with novel reconstruction method can be easily performed, and may be feasible for the treatment of proximal gastric cancer.

## Introduction

In Japan and other Asian countries, the frequency of early gastric cancer (EGC) in the upper third of the stomach continues to increase along with advances in diagnostic techniques, mass screening programs, and removal of helicobacter pylori (1, 2). Although total gastrectomy (TG) is often performed for proximal ECG in Western countries, proximal gastrectomy (PG) as well as TG is performed in Japan. Since Uyama et al. have firstly reported laparoscopic proximal gastrectomy (LPG) in 1995 (3), the frequency of LPG is increasing year by year in Japan. Surprisingly, according to the 14th nationwide survey of endoscopic surgery in Japan by the Japan Society of Endoscopic Surgery, LPG for gastric cancer has been performed in approximately 4,700 cases during the 10-year period from 2008 to 2017, which has increased approximately 6 times compared with the past 10 years (4). The reason for the increased number of LPG is that the benefits of PG over TG include the prevention of postoperative weight loss and undernutrition by preserving the gastric function (5, 6). Therefore, LPG is classified as an optional treatment for proximal EGC in Japanese gastric cancer treatment guidelines (7).

Although PG is theoretically superior procedure for upper one-third of EGC over TG, PG is not the standard procedure worldwide. As problems of PG, the high frequency of anastomosis-related complications such as anastomotic leakage and stenosis, and reflux symptom are reported (8–10). Especially, the high incidence of reflux esophagitis after PG is the fateful problem that is hard to be solved, because of the removal of anti-reflux mechanism including His angle and the lower oesophageal sphincter by PG. To improve these problems, a variety of reconstructive techniques following a PG have been developed to date (11–15). However, the reconstructive method of PG has not been standardized yet. We devised the reconstruction method using the narrow gastric tube in open PG in 1999 (16). And, we have applied this method (conventional method) to the laparoscopic surgery in 2006. For more decreased incidence of reflux esophagitis, we have improved the esophagogastric reconstruction by using long and narrow cobra head-shaped gastric tube (novel method) in 2009 (17).

This study aimed to clarify the safety and feasibility of LPG with the novel method in the comparison with those of LPG with our conventional method from the viewpoint of short-and long-term outcomes.

## Methods

### Patients

**Figure 1:**
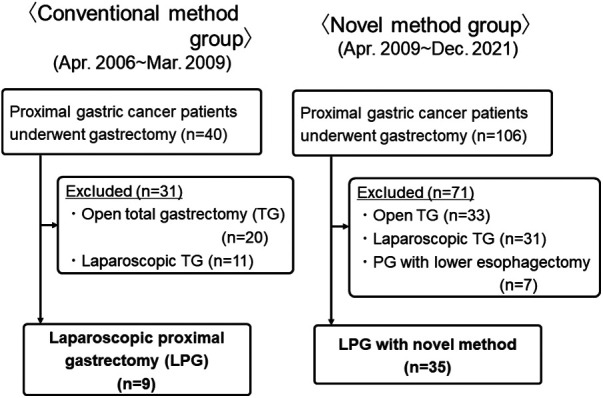
The flowchart of the patients in this study.

From April 2006 to December 2021, a total of 44 patients whom LPG was performed at our department were enrolled in this study. Nine patients underwent LPG with conventional method between April 2006 and March 2009 (Conventional group). And, 35 patients underwent LPG with novel method between April 2009 and December 2021 (Novel group) (Figure 1). Diagnosis of all patients was made according to preoperative endoscopy, endoscopic ultrasonography, upper gastrointestinal series, and abdominal computed tomography (CT). The staging of the tumor was classified according to the Japanese Classification of Gastric Carcinoma by the Japanese Gastric Cancer Association, 3rd English edition (7). Our indication for this LPG is proximal gastric cancer, for which the preoperative diagnosis is clinical T1N0M0, T1N1M0 or clinical T2N0M0 gastric cancer located in the upper one- third of the stomach without esophageal invasion, and it was considered that at least two-thirds of the stomach could be preserved preoperatively in all cases. Differentiated types included papillary and tubular adenocarcinomas, and undifferentiated types included poorly differentiated adenocarcinoma, signet ring cell carcinoma, and mucinous adenocarcinoma. All tissues were examined by expert pathologists.

### Surgical procedure of LPG with long and narrow gastric tube reconstruction: novel method

**Figure 2:**
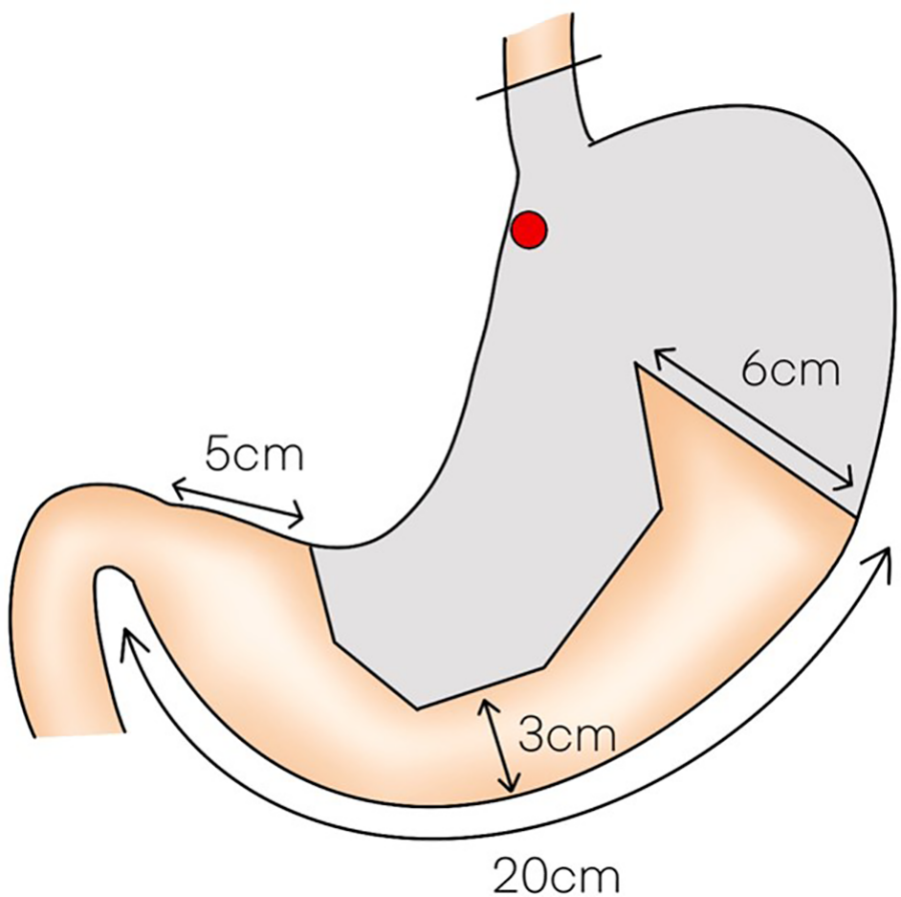
Schematic of the formation of a long and narrow gastric tube like a cobra's head.

**Figure 3:**
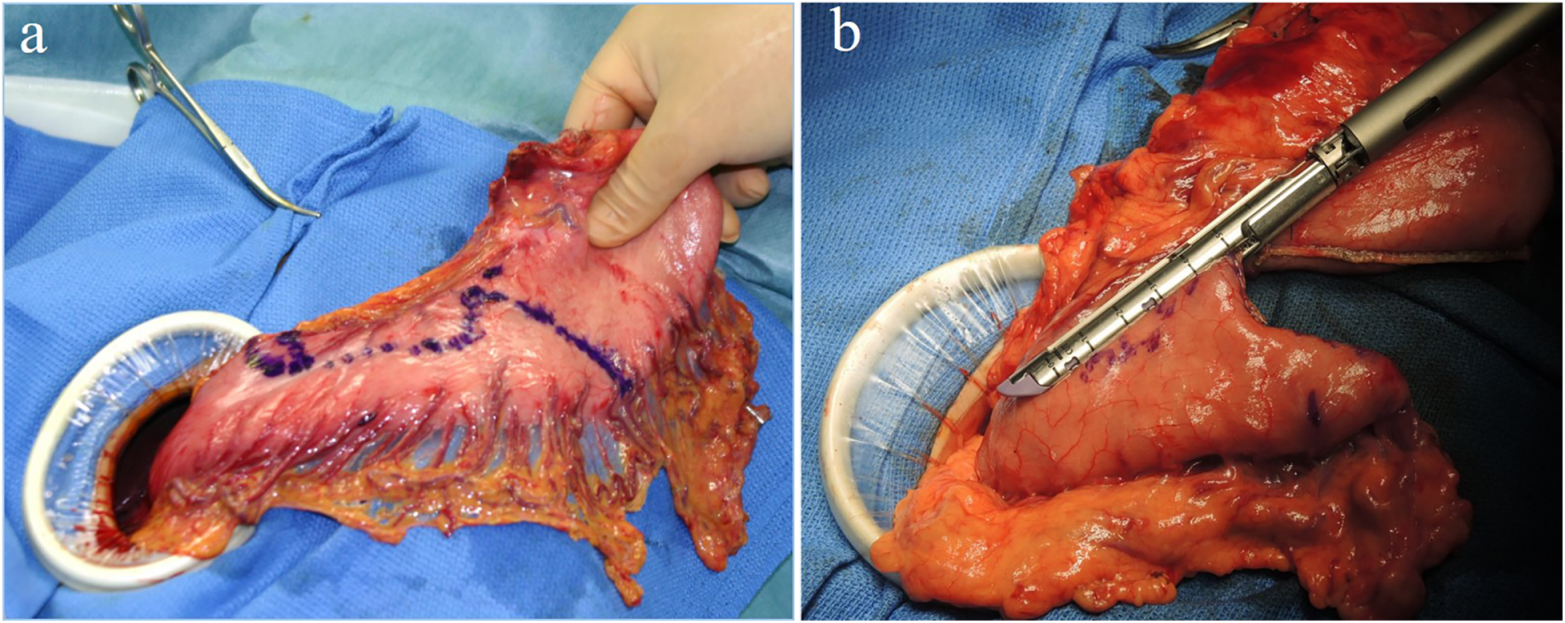
(**A,B**) A long and narrow gastric tube like a cobra's head is created by using linear stapler.

**Figure 4:**
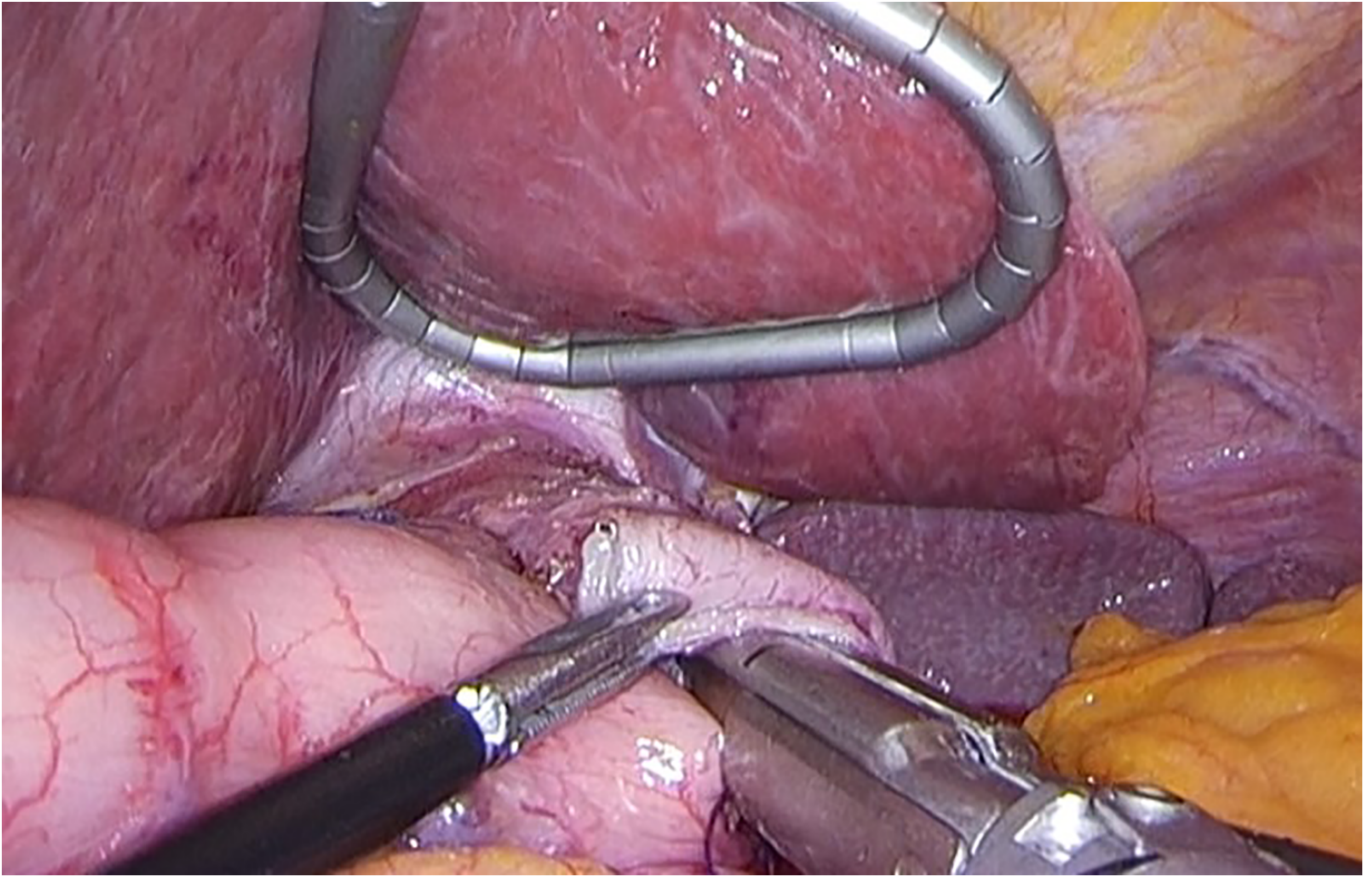
Direct anastomosis with overlap method between the posterior wall of the esophagus and anterior wall of the gastric tube using a 45-mm linear stapler.

**Figure 5:**
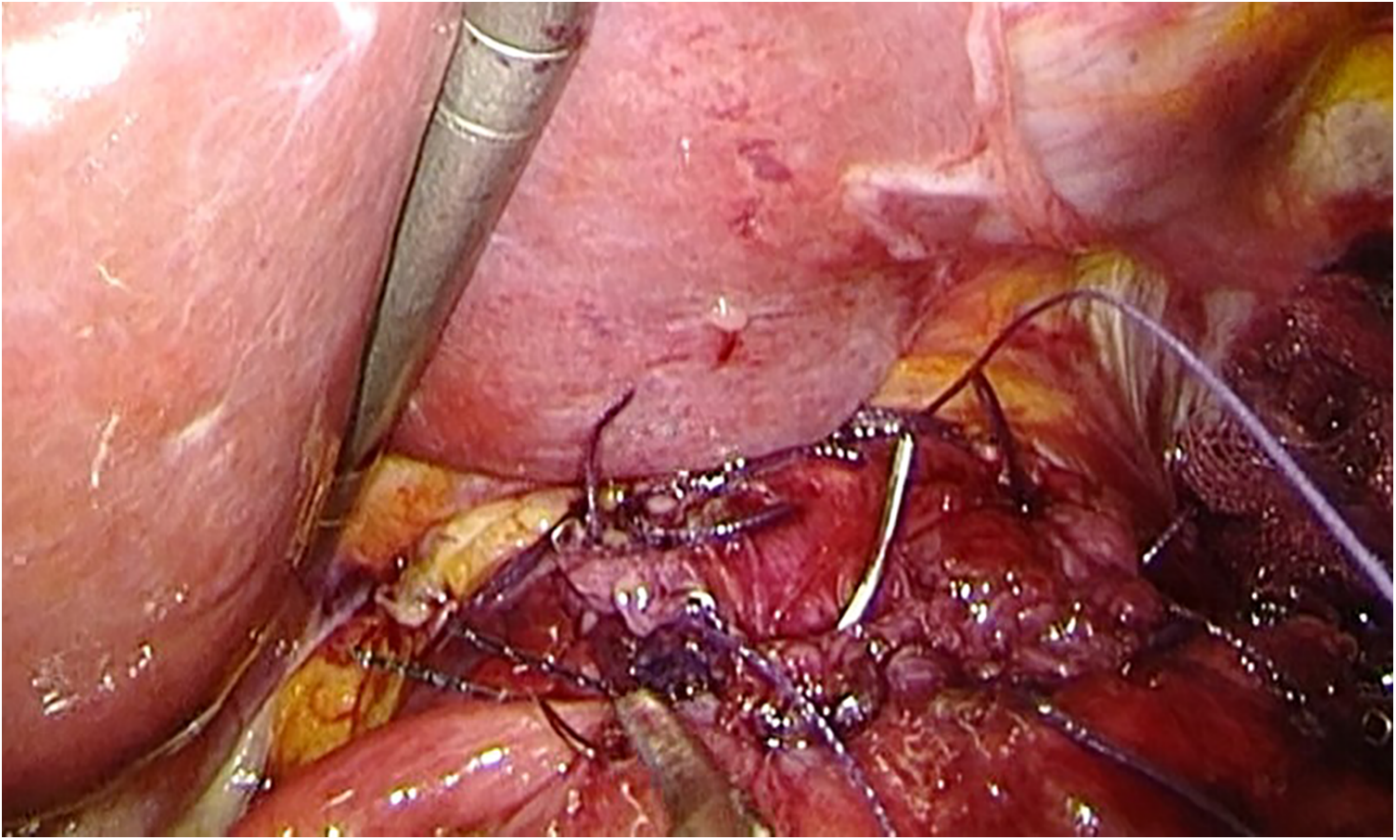
The right ends of the esophageal wall are fixed to the gastric wall.

**Figure 6:**
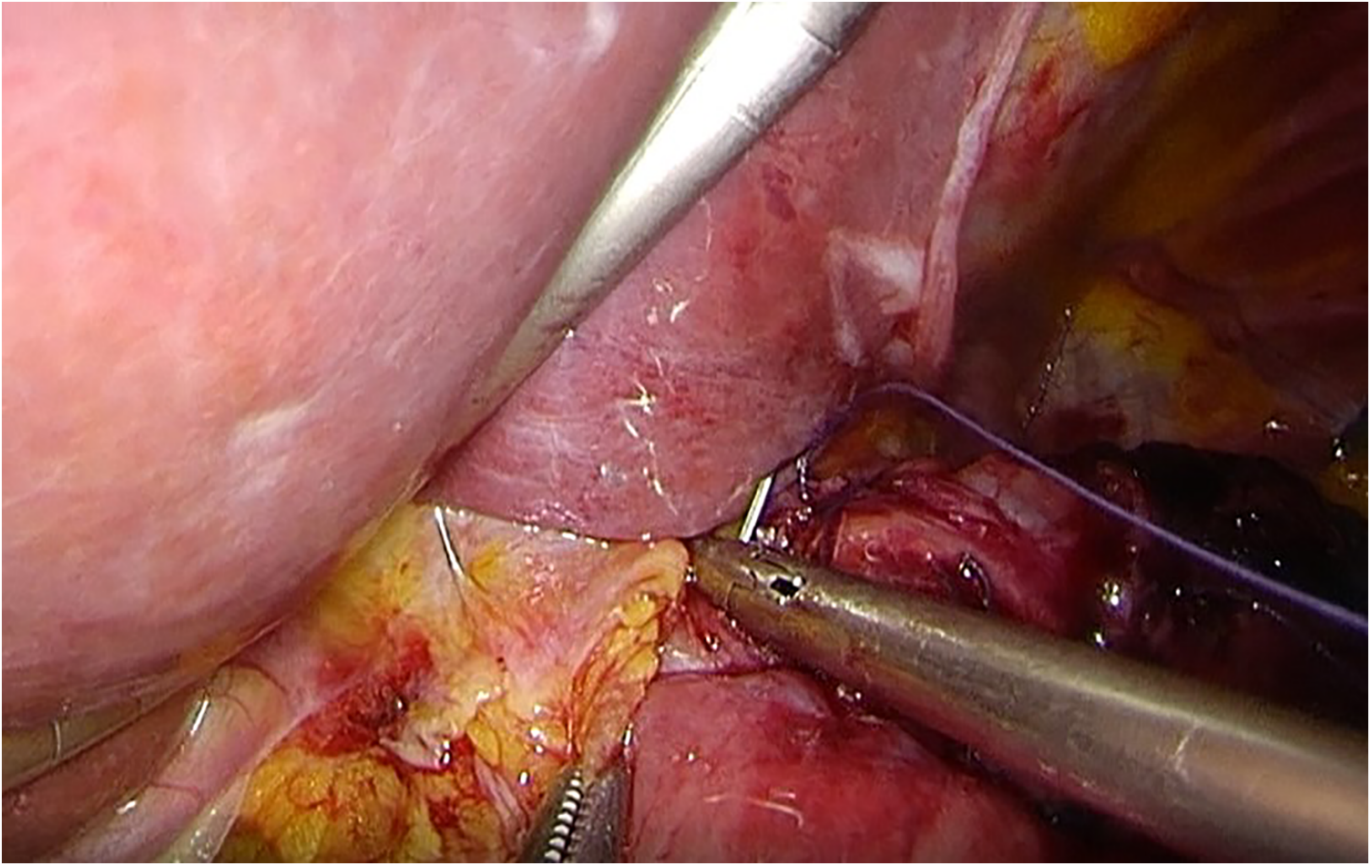
The suture fixation between right crus of diaphragm and esophagus wall.

**Figure 7:**
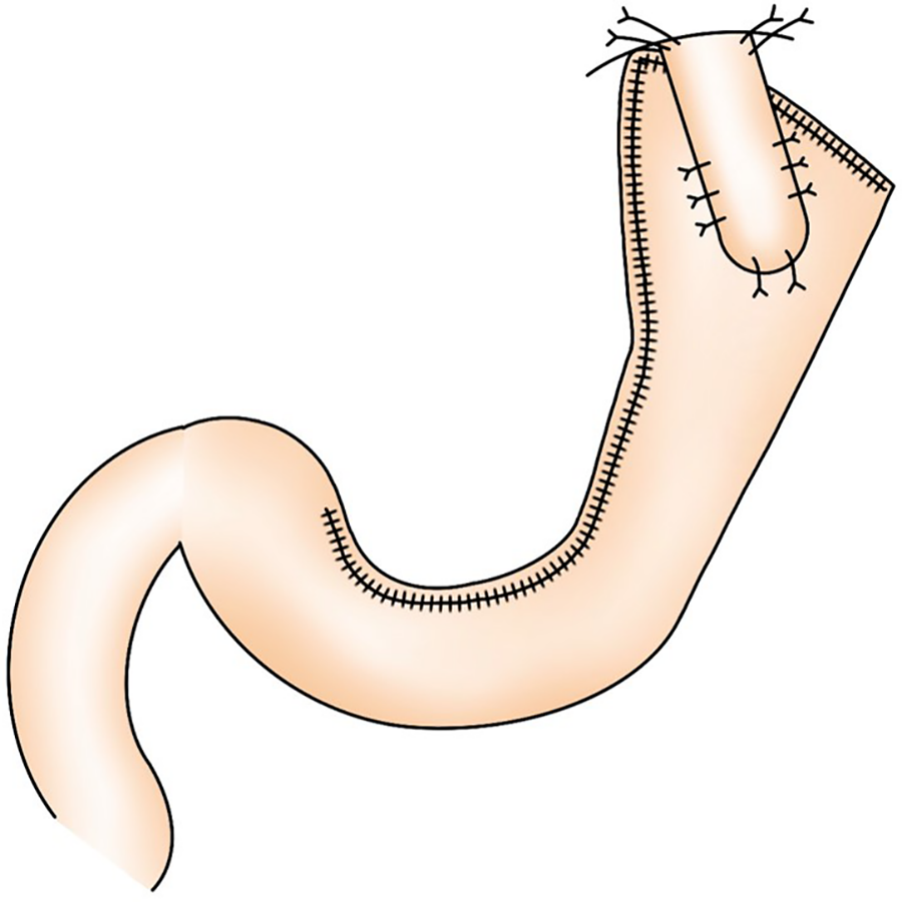
Schematic of the completion of a novel reconstruction method.

LPG with D1 + lymph nodes dissection (nodes no. 1, 2, 3a, 4sa, 4sb, 7, 8a,9, and 11p) were performed according to the Japanese gastric cancer treatment guidelines 2018 (5th edition) (7). During the laparoscopic procedure, the upper part of the stomach is fully mobilized with perigastric and suprapancreatic lymph nodes dissection. We always preserve the vagus nerves, especially, the hepatic and the peripheral pyloric branches. And then the abdominal esophagus is transected. After a mini-laparotomy is created, the entire stomach is pulled outside. A long and narrow gastric tube (more than 20 cm in length, 3 cm width) with widening of the proximal side (6 cm in length) of the gastric tube like a cobra's head is created using by linear stapler (Figures 2, 3). A cobra's head gastric tube is made for formation of the pseudo-fundus. A pyloroplasty is not performed. After the pneumoperitoneum is recreated, esophagogastrostomy is performed by direct anastomosis with overlap method between the posterior wall of the esophagus and anterior wall of the gastric tube using a 45-mm linear stapler under laparoscopic view (Figure 4). At this point, the stapler is inserted toward the right apex of the cobra's head to increase the contact area between esophagus and remnant stomach. The entry hole for anastomotic stapler is closed with a continuous suture by synthetic absorbable barbed threads. To prevent esophageal reflux, both the right and left ends of the esophageal wall are fixed to the gastric wall with laparoscopic interrupted sutures (Figure 5). In addition, we always perform the suture fixation between bilateral crus of diaphragm and esophagus wall for preventing the torsion of the gastric tube (Figures 6, 7).

### Surgical procedure of LPG with gastric tube reconstruction: conventional method

As for all cases in LPG with conventional method, direct anastomosis between the esophagus and gastric tube by a circular stapler was performed. After the upper part of the stomach was fully mobilized, the abdominal esophagus was transected. The stomach was cut between the points of the distal three fourths of the lesser curvature and a half of the greater curvature, and a long gastric tube measuring 15 cm in length and 4 cm in width was made. The lower esophagus is anastomosed to the posterior wall of the gastric tube with a circular stapler inserted through a small opening made on the anterior wall of the stomach. Direct anastomosis between the esophagus and gastric tube was completed.

### Evaluation of short- and long-term outcomes

Short- and long-term outcomes of 35 patients who underwent LPG with novel method were compared with those of 9 patients who underwent LPG with conventional method. We examined the following clinicopathological characteristics of all patients undergoing LPG, such as age, sex, body mass index (BMI), clinicopathological findings including tumor size, clinical TNM factors, pathological TNM factors. Surgical findings, such as operation time and blood loss, were also examined. We also examined the following data to evaluate the short- and long-term outcomes, such as postoperative mortality, start of diet food, postoperative hospital stay, postoperative complications defined as any condition requiring conservative or surgical treatment occurring within 30 days after the operation, including anastomotic leakage, anastomotic stenosis, pancreatic fistula, stasis, and postoperative general complications including respiratory, cardiovascular, and renal disorders, and enterocolitis. Postoperative complications were classified according to the Clavien-Dindo (CD) classification (18). Patients were routinely followed-up by clinical visits every 6 months for 5 years at least. They consisted of a clinical examination, blood tests, thoraco-abdominal CT examination. Followed-up endoscopy was routinely performed at 1 year after operation to evaluate reflux esophagitis, classified according to the Los Angeles classification (19).

### Statistical analysis

Quantitative data are given as the median and range. Differences between the two groups were assessed by the chi-square test, Fisher's exact test, or *t*-test as appropriate. A *p*-value of < 0.05 was considered to indicate statistical significance. These analyses were carried out using SPSS ver. 24 (SPSS Inc., Chicago, IL, USA).

## Results

The patients' demographics are presented in Table 1. All patients in the Conventional group had EGC. Whereas 10 patients had clinical T2 tumor in Novel group. Final pathological diagnosis showed p-stage I in all patients in Conventional group. On the other hand, in Novel group, 3 patients had p-stage II tumor and 1 patient had p-stage III tumor. The surgical and short-term outcomes of all patients are shown in Table 2. In the operation time, there were no significant differences between the two groups (Novel vs. Conventional: 307 vs. 269 min). The amount of blood loss in the Novel group was significantly lower than those in the Conventional group (30 vs. 110 ml, *p* < 0.05). The number of harvested lymph nodes in the Novel group were significantly more than those in the Conventional group (19 vs. 12, *p* < 0.05). No cases of conversion to open surgery and intraoperative death were observed in both groups, and 1 case of intraoperative complication (blood transfusion due to intraoperative bleeding) was observed in the Conventional group. Radical operation was performed in all patients of both groups. In the Novel group, postoperative complications were observed in 3 patients (9%, pancreatic fistula, pneumonia, and stasis in each 1 patient). No cases of anastomotic leakage and stenosis were observed in both groups. There were no significant differences in start of diet and postoperative indication between two groups.

**Table 1:** Patients’ demographics.

Variables	Novel group (*n* = 35)	Conventional group (*n* = 9)	*p*
Age (years, mean ± SD)	69 ± 9	62 ± 11	**0** **.** **04**
Gender			
Male	27	5	0.23
Female	8	4	
BMI (kg/m^2^, mean ± SD)	22.7 ± 3.2	23.4 ± 3.2	0.59
Previous treatment with ESD	13 (37%)	2 (22%)	0.70
Clinical diagnosis			
Clinical T stage (T1a/T1b/T2)	11/14/10	5/4/0	0.20
Clinical N stage (N0/N1)	31/4	9/0	0.57
Clinical stage (IA/IB/II)	25/6/4	9/0/0	0.33
Tumor size (mm, mean ± SD)	27 ± 17	31 ± 14	0.52
Pathological diagnosis			
Histological type			
Differentiated/Undifferentiated	29/6	6/3	0.36
Pathological T stage (T1/T2/T3/T4)	25/6/3/1	7/2/0/0	0.08
Pathological N stage (N0/N1/N2)	30/3/2	8/1/0	0.59
Pathological stage (IA/IB/II/III)	25/6/3/1	7/2/0/0	0.08

SD, standard deviation; BMI, body mass index. The bold means significant difference.

**Table 2:** Surgical and short-term outcomes of patients undergoing LPG.

Variables	Novel group (*n* = 35)	Conventional group (*n* = 9)	*p*
Operation time (min, mean ± SD)	307 ± 58	269 ± 58	0.09
Blood loss (ml, median) (range)	30 (3–220)	110 (5–3,210)	**0** **.** **03**
Harvested lymph nodes (mean ± SD)	19 ± 9	12 ± 4	**0**.**02**
Conversion to open surgery	0	0	
Intraoperative complication	0	1 (blood transfusion)	0.21
R0 resection	35 (100%)	9 (100%)	
Mortality	0	0	
Postoperative complications	3 (9%)	1 (11%)	0.81
Anastomotic leakage	0	0	
Anastomotic stenosis	0	0	
Pancreatic fistula	1 (3%)	0	
Pneumoniae	1 (3%)	1 (11%)	
Stasis	1 (3%)	0	
Start of diet (days, mean ± SD)	5 ± 4	5 ± 2	0.75
Postoperative hospital stay (days, mean ± SD)	19 ± 10	15 ± 3	0.27

SD, standard deviation. The bold means significant difference.

The data of long-term outcomes are summarized in Table 3. All patients received a postoperative endoscopy at 1 year after operation. Reflux esophagitis of grade B or higher of the Los Angeles classification was observed in 6 patients in novel group, and 4 patients in conventional group (17% vs. 44%). The cases of postoperative reflux esophagitis in the Novel group were less than those in the Conventional group, but not significantly. All of these patients were well-controlled by medication of proton-pump inhibitor only. During the median follow-up of 1,270 (range 13–3,868) days, postoperative recurrence was observed in 2 patients with advanced proximal gastric cancer (stage IIB and Stage IIIC) (7%) in the Novel group. No postoperative recurrence was observed in the Conventional group.

**Table 3:** Long-term outcomes of patients undergoing LPG.

Variables	Novel group (*n* = 35)	Conventional group (*n* = 9)	*p*
Postoperative endoscopic findings at 1 year after operation			
Stenosis	0	0	
Reflux esophagitis (LA grade B or higher)	6 (17%)	4 (44%)	0.14
Recurrence	2 (6%)	0	0.31

LA, Los Angeles.

## Discussion

In this study, we compared the clinical outcomes between our novel method and conventional method following LPG. All patients in this study were completed in curative resection by LPG. In comparison between the Novel and Conventional group, the Novel group tended to have more advanced cancer patients. There were no differences in the operation time and the incidence of postoperative complications between the two groups, whereas the amount of blood loss in the Novel group was less than those in the conventional group. Postoperative anastomosis-related complications such as leakage and stenosis did not occur in this study. Although there was no significant difference, the frequency of postoperative reflux esophagitis in the Novel group was lower than those in the Conventional group. The LPG with our novel reconstruction method for proximal EGC is a simple, safe technique that may prevent anastomosis-related complications.

Regarding the surgical treatment for EGC in the upper third of the stomach, the following three issues have been mainly discussed; the first issue is the operation method, TG or PG. The second is the reconstruction method following PG. The third is the oncological safety in PG.

In the Western countries, TG is more often performed for proximal ECG. The following concerns may be the reason why PG is considered to inferior to TG for proximal gastric cancer. The first concern is whether the quality of life after PG is not better than after TG, regarding loss of body weight and suffering malnutrition. The second is the occurrence of postoperative anastomosis-related complications including, anastomotic leakage and stenosis following PG. The third is the occurrence of severe reflux esophagitis after PG. Therefore, many surgeons in the Western countries have chosen to perform TG even for upper-third EGC. To improve these concerns, various reconstruction procedures following PG have been developing, especially in Japan. Until now, three reconstruction methods, including double-tract reconstruction (DT), jejunal interposition (JI), and esophagogastrostomy, have been popularly performed following PG in Japan. However, a standard reconstruction procedure following PG has not been established because each procedure has some disadvantages. Recently, Zhang et al. demonstrated that DT is superior to esophagogastrostomy in controlling reflux esophagitis after PG from a prospective randomized controlled clinical trial in China. DT may indeed be superior to our reconstruction method in preventing esophagitis. However, our reconstruction method is easier and less complicated, and may have fewer postoperative anastomotic complications than DT (20).

Esophagogastrostomy is the simplest reconstruction procedure than other procedures, DT and JI. However, esophagogastrostomy have a high risk of reflux esophagitis and anastomotic stenosis. Therefore, several procedures for preventing reflux esophagitis in esophagogastrostomy have been reported. Okabe et al. reported the reconstruction method with an esophagogastrostomy using a knifeless linear stapler and hand suturing (21). This procedure is similar to our method in that the use of linear stapler and the fixation of the esophagus onto the anterior wall of the remnant stomach. However, their procedure is end-to-side anastomosis by continuous hand suturing. Hand suturing of anastomosis is likely the cause of anastomotic stenosis. Yamashita et al. reported a new method of esophagogastrostomy, side overlap with fundoplication by Yamashita (SOFY) (22). They performed side overlap esophagogastrostomy by linear stapler rotated counterclockwise on its axis, suturing the gastric wall to the left side of the esophagus. This method is also easily procedure like our method, but concerns about reflux still remain due to the patients' position. Kamikawa et al. described a novel esophagogastrostomy procedure with double-flap technique (23), and this procedure was applied to LPG (24–26). Although the short-term outcomes regarding reflux prevention following these procedures have been reported to be satisfactory, these techniques are very complicated. So, these challenging techniques may prevent the widespread use of LPG for upper one-third of EGC.

As shown in the present study, our novel procedure is so simple and can be easily and safely performed by younger surgeons who are familiar with the laparoscopic standard technique. Our novel reconstruction method are characterized by the following features: (1) reconstruction by a long and narrow gastric tube with the sufficient capacity; (2) making a pseudo-gastric angle by long gastric tube for staying food in the gastric tube to some degree; (3) a pseudo-fundus by making gastric tube with widening of the proximal side like a cobra's head; (4) preserving the excretory function of residual stomach by avoidance of No.5 and No.6 node dissection around the pyloric ring; (5) the anastomosis by on-lay method with tight suturing between the esophageal muscularis fascia and the gastric tube flatten the esophagus; (6) fixing the esophagus to bilateral crus of diaphragm for the prevention of twisting and lifting of the esophageal stump. In this study, reflux esophagitis of grade C was observed in only 4 patients (11%). These results might be caused by our novel reflux prevention systems. Besides, we noted no other anastomosis-related complications during this study. These results suggest that our novel reconstruction method is a feasible procedure as the reconstruction following PG. On the other hand, we consider that the incidence of postoperative reflux esophagitis in our novel method is still high compared to other methods, and our novel methods are inadequate for preventing postoperative reflux esophagitis. Therefore, we believe that our novel methods still need to be improved to prevent reflux esophagitis.

Several studies have concluded that long-term outcomes of open PG for proximal ECG were not shown to be different from those of open TG (10, 27, 28). Oncological safety in LPG might have not become a major concern due to these previous results. Thus, there is little evidence of oncological safety of LPG. Ahn et al. reported that overall survival for proximal gastric cancer was similar when comparing LPG and LTG (9). In our study, recurrence was observed in only 2 patients with advanced proximal gastric cancer. We have been expanding operative indication from early to advanced gastric cancer, recently. We consider that LPG may be an oncologically acceptable procedure for stage I and IIA at least. Large sample size of patients is necessary to confirm these considerations.

The present study has some limitations. First, this retrospective study is of little value and significance because of very small sample size, especially, conventional method. Second, historical bias in this study could be an element of weakness due to many changes in terms of surgical technique (e.g., materials) and its standardization that may have affected the outcomes over conventional method. Third, the nutritional status and quality of life of patients in both groups were not examined in this study. It will be necessary to carry out the long-term nutritional evaluation in the near future. Third, the comparison of outcomes did not include TG. Further examination and longer follow up of patients are needed.

## Conclusions

In conclusion, LPG with long and narrow gastric tube reconstruction method can easily performed, and may be feasible for the treatment of gastric cancer in the upper third of the stomach because of safe technique for preventing anastomosis-related complications. However, we consider that there is still room for improvement with regard to prevent reflux esophagitis in even with our novel methods.

## Data Availability

The raw data supporting the conclusions of this article will be made available by the authors, without undue reservation.
